# Preparation of Hot-Pressed Wheat Straw Board by Self-Adhesive Process: Effects of Raw Material Sizes and Acid/Alkali Pretreatment

**DOI:** 10.3390/ma17235845

**Published:** 2024-11-28

**Authors:** Jianing Wang, Ziyue Feng, Jiachen Zuo, Qinzhen Fan, Libo Zhang

**Affiliations:** 1 Guangdong Provincial Engineering & Technology Center for Corrosion and Safety in Petrochemical Industry, School of Chemical Engineering, Guangdong University of Petrochemical Technology, Maoming 525000, China; wangjianing62022@163.com (J.W.); zfengar@connect.ust.hk (Z.F.); zjcenormous@outlook.com (J.Z.); 2 State Key Laboratory of Heavy Oil Processing, College of Engineering, China University of Petroleum-Beijing at Karamay, Karamay 834000, China

**Keywords:** wheat straw, size effect, acid/alkali pretreatment, hot pressing, self-bonding

## Abstract

The development of wheat straw boards utilizing intrinsic bonding mechanisms not only facilitates the high-value utilization of agricultural solid waste but also diminishes the reliance on synthetic adhesives. In this study, using wheat straw as the primary substrate, we investigated the effects of mechanical smashing combined with pretreatment using inorganic acids or alkalis on the properties of hot-pressed boards, as well as the relationship between the properties of hot-pressed boards and the physical properties and chemical composition of wheat straw raw materials. These selective pretreatments effectively degraded lignin, hemicellulose, and other components, thereby promoting fiber reorientation and resulting in a denser microstructure with improved self-bonding capabilities. The optimal board was fabricated with a granularity of 0.3 mm and underwent alkali pretreatment, achieving a tensile strength of 11.564 MPa, an internal bonding strength of 0.556 MPa, and bending strength and modulus of 24.306 MPa and 2.766 GPa, respectively. These findings have significant implications for advancing manufacturing processes and conceptualizing binder-free boards derived from agricultural residues.

## 1. Introduction

Wood-based boards play a crucial role in construction and manufacturing, with a global market value of $175.1 billion in 2023 [1]. These boards, created through the hot pressing of wood or other lignocellulosic biomass, often utilize adhesives to improve their structural integrity.

The conventional production of wood-based boards primarily involves using wood and adhesives, such as urea-formaldehyde (UF), phenol-formaldehyde (PF), or melamine-urea-formaldehyde (MUF), to improve performance [2,3]. While utilizing wood as a raw material leads to high-quality products, it also results in extensive tree cutting, contributing to various environmental issues, like land desertification and global warming [4]. Although these adhesives enhance the boards’ dimensional stability and physicochemical properties, their use has significant downsides. Specifically, these adhesives can emit harmful substances, such as formaldehyde, during the board’s lifespan, leading to environmental pollution and potential health hazards for humans [5,6].

While there has been research into natural adhesives, like lignin, tannin, protein, and starch [7,8,9], the complexity of their production processes and the high cost of raw materials lead to increased manufacturing expenses for boards. As a result, there is growing interest in using non-wood lignocellulosic biomass as alternatives to wood for the production of adhesive-free boards [10].

In recent years, there has been increasing interest in using agricultural waste, such as straw, as raw material for producing non-adhesive hot-pressed boards. However, straw generally has a higher ash content, including silica and wax, compared to wood [11]. As a result, boards made from straw often fall short of the rigidity, bending resistance, and other mechanical properties of those made from wood. For instance, Xiao et al. investigated the effects of treating reed straw with 2% NaOH for hot-pressed board production, achieving an elastic modulus (MOE) of 2486 MPa and a flexural strength of 18.2 MPa [12]. Meanwhile, Shusaku Asano et al. produced hot-pressed boards by pretreating rice husks with hot compressed water, resulting in tensile and flexural strengths of 26 MPa and 21 MPa, respectively [13]. Ireen Parvin Nitu et al. prepared hot-pressed jute boards using mechanical smashing and citric acid pretreatment, achieving an elastic modulus (MOE) of 3774 MPa and a flexural strength of 17.01 MPa [14]. Additionally, Ramunas Tupciauskas et al. created hot-pressed boards from wheat straw and hemp straw through steam explosion pretreatment, yielding an elastic modulus (MOE) of 2750 MPa and a flexural strength of 15.5 MPa [15].

These studies show that pretreating straw can significantly modify the surface morphology of the raw wood fiber biomass, enhancing board performance. Additionally, some research has investigated mechanical smashing, a technique that applies mechanical force to break down raw materials into smaller particles. This reduction in particle size increases the specific surface area, which in turn improves the materials’ self-adhesive properties [16]. However, there is a lack of comprehensive comparative studies examining the effects of various preprocessing methods.

In recent years, wheat has emerged as a crop of considerable economic significance, ranking among the most widely distributed and extensively cultivated and holding the second position in global grain production [17]. In 2023, global wheat production reached an impressive 786 million tons [18]. Notably, wheat straw, which is an agricultural by-product, represents 66.7% of the total weight of wheat. However, conventional methods for utilizing wheat straw—such as returning it to the field, producing fodder, fermenting it for biogas, or using it as fuel—provide limited added value and conversion efficiency [19]. In contrast, converting wheat straw into board products through hot pressing allows for complete utilization of its components, thereby enhancing both economic and social value.

In this context, this study focuses on wheat straw as a raw material, investigating how combined mechanical smashing and inorganic acid (or alkali) treatments affect the properties of hot-pressed wheat straw boards. Building on previous research [20], this study assessed the impact of particle sizes (0.5 mm, 0.3 mm, and 0.1 mm) of mechanically smashed wheat straw on board performance. The results indicate that smaller particle sizes improve water resistance, while the mechanical properties of the boards initially increase before decreasing.

Further treatment of wheat straw particles of different sizes with diluted acid and alkali enhanced the physicochemical properties of the hot-pressed boards. Notably, boards made from 0.3 mm-sized wheat straw particles treated with alkali demonstrated the best mechanical performance. Mechanical processing effectively reduces raw material size, aiding in the separation of components such as cellulose and lignin. Additionally, alkali treatment significantly degrades a large portion of lignin, some hemicellulose, and other components in the wheat straw, exposing more cellulose and creating additional chemically reactive sites (hydroxyl groups). During hot pressing, these reactive sites interact with the exposed functional groups on cellulose, facilitating the formation of new chemical bonds. This interaction causes the wheat straw fibers to realign and create a denser microstructure, thereby increasing the self-adhesiveness of the boards.

## 2. Materials and Methods

### 2.1. Material

Wheat straw was sourced from a farm in Urumqi and initially crushed before being thoroughly washed with water. The straw particles were then classified into different sizes (0.5 mm, 0.3 mm, and 0.1 mm) using laboratory sieves. After sieving, the straw particles were dried at 105 °C for 12 h and stored in sealed bags for future use. The chemicals used, including 0.5 M dilute hydrochloric acid, sodium hydroxide, and anhydrous sodium sulfite, were obtained from China Innochem Chemical Reagents Co. (Beijing, China). Deionized water was prepared using a WPUP-UV-20 system from Chengdu Water Science and Technology Co., Ltd. (Chengdu, China). All reagents were used as received without further purification.

### 2.2. Preparation of Hot-Pressed Wheat Straw Boards

**Figure 1:**
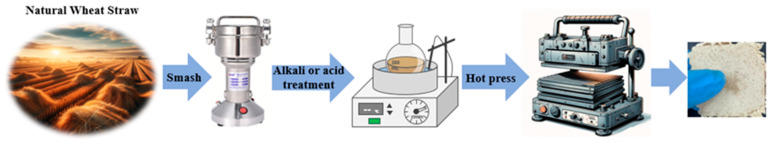
Experimental process for preparing hot-pressed wheat straw boards with different pretreatment methods and particle sizes.

The experimental process of this project is shown in Figure 1. The photographs of boards prepared in this study are shown in Figure S1 of Supporting Information.

Wheat straw powders of varying particle sizes (0.5 mm, 0.3 mm, and 0.1 mm) were subjected to pretreatment in either a 0.5 M hydrochloric acid solution or a mixed alkaline solution comprising 2.5 M NaOH and 0.5 M Na_2_SO_3_ at 95 °C for 4 h. Following pretreatment, the samples were vacuum-filtered, and the residual solids were dried in an oven at 105 °C to achieve constant weight. A consistent 6.5 g of each pretreated wheat straw sample was then placed in a hot-press mold and subjected to a pressing protocol at 160 °C and 15 MPa for 2 h, yielding composite boards with an approximate thickness of 5.0 mm.

The composite boards produced by hot pressing the untreated wheat straw of particle sizes 0.5 mm, 0.3 mm, and 0.1 mm were designated as WS-5, WS-3, and WS-1, respectively. Boards obtained from hot pressing the wheat straw that was pretreated with dilute acid (0.5 M HCl) and had particle sizes of 0.5 mm, 0.3 mm, and 0.1 mm were designated as WS-Acid-5, WS-Acid-3, and WS-Acid-1, respectively. Similarly, boards produced from hot pressing the wheat straw pretreated with the alkaline solution (2.5 M NaOH and 0.5 M Na_2_SO_3_) were denoted as WS-Alkali-5, WS-Alkali-3, and WS-Alkali-1 for the respective particle sizes.

### 2.3. Characterization

#### 2.3.1. Mechanics Performance Testing

Following the GB/T 4897-2015 standard [12], a digital display single-arm microcontrolled electronic universal testing machine (STX1000, EAST, Xiamen, China) was employed to evaluate the tensile strength, bending strength, bending modulus, internal bond strength (IB), and density of each sample group. Five replicates were tested, and the average values were determined. Samples measuring 150 ± 1 × 50 ± 1 × 5.0 mm were used for the tensile and bending tests, while samples sized 50 ± 1 × 50 ± 1 × 5.0 mm were prepared for the internal bond strength test. The samples were securely bonded to the grips using hot-melt adhesive, which was fully cured prior to conducting the tension tests [21].

#### 2.3.2. Morphology Analysis

Each set of samples was dried and mounted on the sample stage using carbon tape. The samples were then sputter-coated with gold under vacuum conditions and placed into the chamber of an environmental scanning electron microscope (Quanta 200, FEI (SEM, Hillsboro, OR, USA)). The voltage was set to 15 kV, and the imaging parameters were adjusted accordingly. The samples were magnified 200 times, and a suitable field of view was selected for surface observation of the board samples. The images were recorded and saved for further analysis.

#### 2.3.3. Water Adsorption of Board

The prepared samples were cut into standard specimens with dimensions of 10 ± 1 × 10 ± 1 mm, and their weights were recorded. In accordance with the Chinese standard GB/T 4897-2015 [12], the conditioned samples were immersed in water at room temperature for 24 h, then towel-dried to remove any surface moisture, and weighed again. Five replicates were tested for each sample group, and the average water absorption rate was calculated.

#### 2.3.4. Fourier Transform Infrared (FTIR)

Fourier transform infrared spectroscopy (FTIR) analysis was conducted using a Thermo Scientific iN10 (Thermo, Waltham, MA, USA), covering the wavenumber range of 600–4000 cm^−1^ [22], in transmittance mode.

#### 2.3.5. X-Ray Photoelectron Spectroscopy (XPS) Analysis

X-Ray photoelectron spectroscopy (XPS) was conducted using a Thermo Scientific K-Alpha instrument (Thermo, Waltham, MA, USA), which features a monochromated Al Kα X-Ray source (hv = 1486.6 eV) operating at a power of 150 W. The chemical composition of the boards prepared using various pretreatment methods was analyzed following the procedures outlined by the National Renewable Energy Laboratory (NREL).

#### 2.3.6. X-Ray Diffraction (XRD) Analysis

X-Ray diffraction (XRD) analysis was performed using a Rigaku SmartLab SE (Rigaku) from Tokyo, Japan, equipped with a Cu Kα radiation source. The scanning angle was set between 5° and 40°, with a scan speed of 2°/min. The crystallinity index (CrI) was determined using the Segal equation, with the calculation formula provided below:

(1) $$CrI\%=\left(\left(I_{200}-I_{am}\right)/I_{200}\right)\times 100$$

where CrI is the crystallinity index, $I_{200}$ is the maximum intensity of the peak near 2θ = 22.5°, and $I_{am}$ is the minimum intensity between the (200) and (110) peaks (around 2θ = 18°). The crystallinity represents the proportion of amorphous cellulose.

#### 2.3.7. Thermogravimetric Analysis (TGA)

The thermal stability of the boards was assessed using thermogravimetric analysis (TGA). Approximately 5 mg of each sample was heated at a rate of 5 °C/min under a nitrogen flow (purity > 99.9999 vol%) rate of 300 mL/min, within a temperature range of 30–700 °C [13]. The TGA measurements were performed using the Hitachi High-Tech Science model (STA7200).

## 3. Results and Discussion

### 3.1. Surface Morphology Analysis

**Figure 2:**
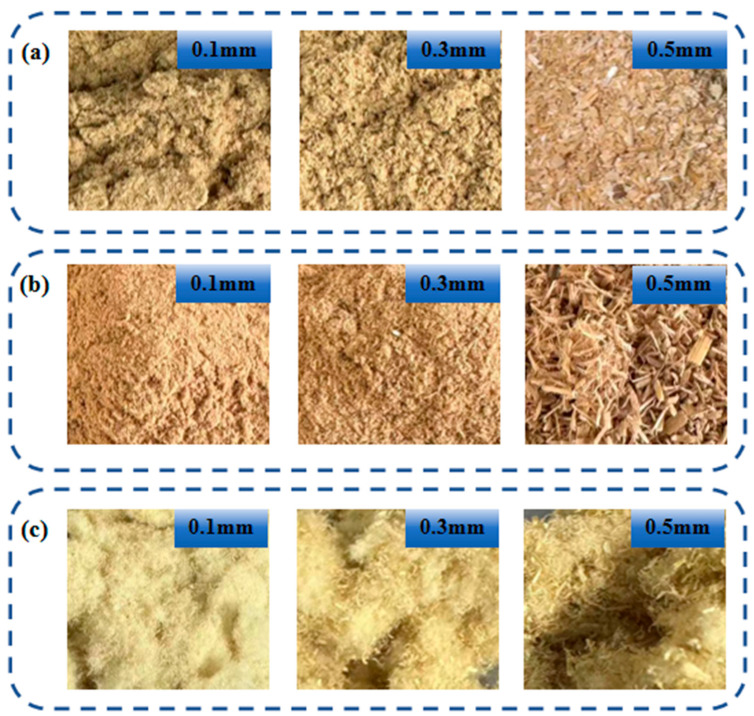
Macroscopic morphology of wheat straw with different pretreatment methods and raw material. (**a**) Untreated wheat straw. (**b**) Wheat straw pretreated with dilute acid. (**c**) Wheat straw pretreated with alkali.

The macroscopic morphology of wheat straw, pretreated using different methods and of varying sizes, is clearly illustrated in Figure 2. A careful analysis and comparison of Figure 2a–c reveals that both dilute acid and alkali pretreatments induce changes in the fluffiness of the wheat straw, albeit to different degrees. Notably, the alkali-pretreated wheat straw demonstrates the most pronounced enhancement in fluffiness.

**Figure 3:**
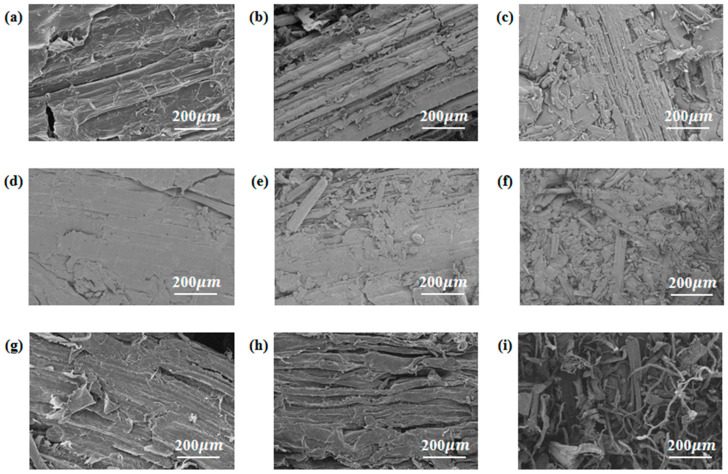
Microscopic morphology of the surface of hot-pressed wheat straw boards with different pretreatments and raw material sizes. (**a**) WS-5. (**b**) WS-3. (**c**) WS-1. (**d**) WS-Acid-5. (**e**) WS-Acid-3. (**f**) WS-Acid-1. (**g**) WS-Alkali-5. (**h**) WS-Alkali-3. (**i**) WS-Alkali-1.

The surface microstructure of hot-pressed wheat straw boards prepared through various pretreatment methods and sizes is vividly depicted in Figure 3. Upon meticulous examination of Figure 3a–c, it becomes apparent that the surfaces of the WS group samples maintain their natural state, exhibiting a rough texture and numerous irregular pores. Figure 3d,f clearly demonstrate that dilute acid pretreatment leads to a smoother surface on the wheat straw boards. This smoother texture is accompanied by a looser arrangement of wheat straw fibers and a higher number of pores compared to the WS group samples. This observation can be attributed to the removal of certain hemicellulose and other components from the wheat straw during the dilute acid pretreatment process [23]. A comparison of Figure 3g,i with the WS samples reveals that the fiber surfaces of the WS-Alkali group samples exhibit a rougher texture, with a more pronounced exposure of cellulose on their surfaces. Additionally, there is a notable increase in porosity between the fibers and a greater dispersion of fibers. This transformation can be attributed to the alkali pretreatment, which effectively removes a significant portion of lignin, along with some hemicellulose, proteins, pectins, and other components from the wheat straw. Furthermore, the increase in the number of hydroxyl groups present on the fiber surfaces enhances the adhesive forces between the fibers, leading to a more cohesive and robust structure.

As the size of the raw material particles diminishes, the board surface becomes smoother. This occurs because smaller particles are able to fill the gaps between fibers more effectively during the hot-pressing process, resulting in a more uniform and smoother surface. Simultaneously, the adhesion between wheat straw particles tightens, and the interfacial gaps are significantly reduced. The increased specific surface area of the raw material particles resulting from their size reduction accounts for the smoother board surface. This enhancement in surface area enhances the self-adhesive capability of the board. When the size of the raw material particles becomes excessively small (0.1 mm), as evident in Figure 3c,f,i, the high density and low porosity associated with these smaller particles manifest macroscopically in the form of a smooth surface devoid of visible gaps. However, this reduction in porosity negatively affects the internal air permeability of the board, compromising its moisture and heat transfer capabilities. Consequently, this leads to an increase in microscopic defects, which, to a certain extent, hinder the optimal performance of the board.

Analysis of SEM characterization images reveals that both dilute acid and alkali pretreatments have induced varying degrees of microstructural alterations in hot-pressed wheat straw boards. Notably, the alkali pretreatment exhibits a particularly profound impact on the microstructural changes observed in these boards. This is due to the fact that, in comparison to dilute acid pretreatment, alkali pretreatment not only removes a portion of the hemicellulose and other components present in wheat straw but also eliminates a significant amount of lignin, leading to a more compact arrangement of wheat straw fibers. Additionally, by decreasing the particle size and enhancing the specific surface area of the particles, a denser microstructure is achieved among the wheat straw fibers. However, excessively small particle size can reduce the porosity of the board, hindering the efficient transfer of moisture and heat during the hot-pressing process. This ultimately leads to an increase in micro defects within the board, thereby compromising its overall performance.

### 3.2. Chemical Structure Analysis

**Figure 4:**
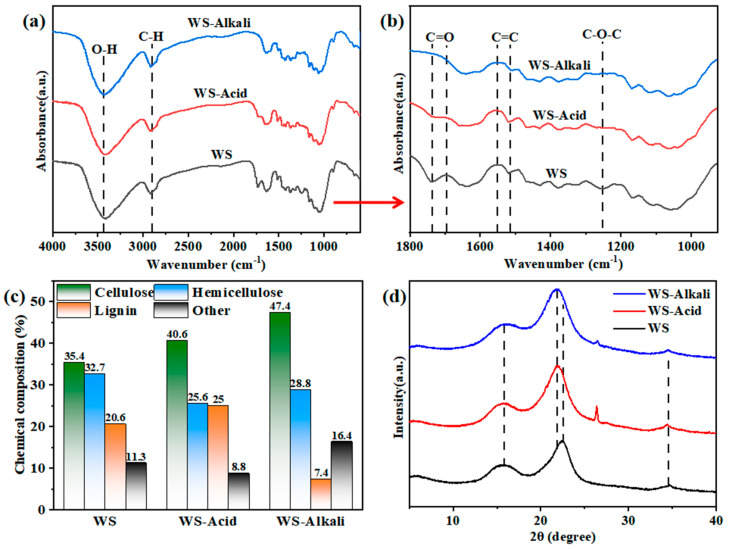
Chemical structure characterization of hot-pressed wheat straw boards with different pretreatments. (**a**,**b**) FTIR spectra. (**c**) Chemical composition graph. (**d**) XRD spectra.

Utilizing FTIR characterization, valuable insights into the functional groups of materials can be gained. Figure 4a,b depicts the FTIR spectra of hot-pressed wheat straw boards that have undergone different pretreatments. Samples of WS-Alkali, WS-Acid, and WS all exhibit a broad peak at 3400 cm^−1^, which is attributed to the stretching vibrations of the O-H bonds. This peak serves as evidence for the presence of the O-H bonds, a characteristic trait of alcohols and phenols, indicating the significant cellulose component found in wheat straw. Additionally, a peak at 2918 cm^−1^ indicates the stretching vibrations of the C-H bonds in alkanes. The absorption peak observed at 1730 cm^−1^ is attributed to the acetyl and C=O stretching vibrations within hemicellulose. Meanwhile, the peak at 1679 cm^−1^ in wheat straw suggests the presence of proteins (amide bonds) or other carbonyl-containing compounds. These peaks are caused by the specific vibrations of chemical bonds within the respective components of wheat straw. Compared to the untreated wheat straw, the intensity of absorption peaks at 1679 cm^−1^ and 1730 cm^−1^ is significantly reduced in wheat straw treated with both alkali and dilute acid. This suggests that both pretreatments degrade hemicellulose and other components to a certain extent. Furthermore, peaks at 1517 cm^−1^ and 1545 cm^−1^, which are attributed to the C=C stretching vibrations of aromatic rings in lignin, and the absorption peak at 1260 cm^−1^, associated with the ether bond (-O-) stretching vibrations of aromatic rings in lignin, exhibit a notable decrease in intensity in the WS-Alkali samples compared to the WS-Acid and WS samples. Spectral analysis indicates that after dilute acid pretreatment, partial removal of hemicellulose and other components occurs. In contrast, alkali pretreatment results in the partial removal of lignin, hemicellulose, proteins, and other components. Both pretreatments effectively expose the functional groups present on the surface of wheat straw cellulose. Under the hot-pressing conditions of 160 °C, 15 MPa, and 2 h, this exposure facilitates the self-adhesion between wheat straw fibers, ultimately enhancing the overall performance of the board.

Figure 4c illustrates the chemical composition of the WS-Alkali, WS-Acid, and WS group samples. The mass lost after acid pretreatment is 5.45 %, and the mass lost after alkali pretreatment is 11.37 %. The comparison with the chemical composition graph of the WS group samples reveals that dilute acid pretreatment effectively removed some hemicellulose and other components from wheat straw, resulting in a decrease in their content in the WS-Acid group samples. On the other hand, alkali pretreatment achieved a more comprehensive degradation, removing most of the lignin along with some hemicellulose and other components, leading to a reduction in their content in the WS-Alkali group samples. Analysis of the chemical composition graphs of hot-pressed wheat straw boards with different pretreatments further confirms that dilute acid pretreatment degraded some hemicellulose and other components, while alkali pretreatment degraded most of the lignin, as well as some hemicellulose and other components. These changes in chemical composition are consistent with the observed patterns in the FTIR spectra.

XRD characterization analysis provides valuable insights into the crystalline structure of the boards. Figure 4d presents the XRD spectra of hot-pressed wheat straw boards treated with various pretreatment methods, offering a comprehensive understanding of their structural properties. There are distinct peaks around 15.8°, 22.5°, and 34.7°, corresponding to the (110), (200), and (004) lattice planes, which are characteristic features of cellulose I [24]. The crystallinity index (CrI) of the WS-Alkali sample stands at 53.4%, marking a notable increase compared to both the WS-Acid samples, which have a CrI of 48.7%, and the WS samples, with a CrI of 43.8%. This is because the alkali pretreatment removed the majority of lignin and some hemicellulose, substantially reducing the amorphous regions, and the hydroxyl groups in the amorphous regions lose water through condensation, forming ether bonds, which also leads to an increase in CrI [25]. Additionally, alkali treatment converts cellulose I to cellulose II [26], which is evident in the XRD spectra, as the characteristic peak of cellulose I around 22.5° shifts to around 22° in the WS-Alkali samples. During the hot-pressing process, the cellulose molecular chains undergo reorganization, resulting in a more stable crystalline structure. The observed increase in the crystallinity index (CrI) value for hot-pressed wheat straw boards treated with dilute acid can be attributed to the dissolution of amorphous regions within the cellulose structure. Specifically, the removal of hemicellulose and other components enables a superior arrangement and bonding of cellulose molecular chains during the hot-pressing process, ultimately enhancing the crystallinity of the boards.

**Figure 5:**
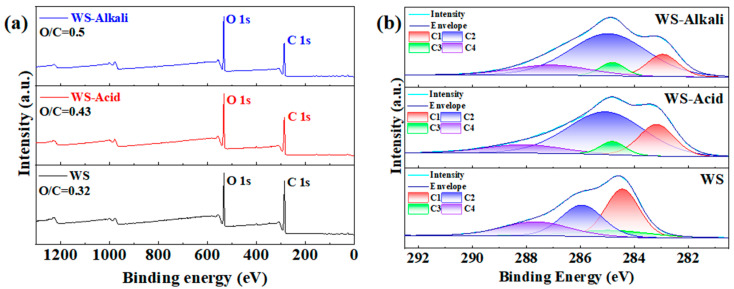
(**a**) X-Ray photoelectron spectroscopy (XPS) spectra. (**b**) C 1s X-Ray photoelectron spectroscopy (XPS) spectra.

XPS characterization, a sophisticated technique, is employed to delve into the surface chemical composition of hot-pressed wheat straw boards, particularly those treated with alkali, dilute acid, and those untreated. Figure 5a presents the XPS spectra of these boards, offering a comprehensive view of their surface chemistry. The surface O/C ratio of the WS-Alkali sample stands at 0.5, while that of the WS-Acid sample is 0.43, both notably higher than the ratio of the untreated WS sample, which is 0.32. This difference can be attributed to the distinct effects of the two pretreatment methods. Alkali pretreatment effectively reduces the concentration of lignin and hemicellulose in wheat straw, resulting in a greater exposure of cellulose on the surface of the WS-Alkali samples. On the other hand, dilute acid pretreatment primarily removes a portion of the hemicellulose without significantly affecting lignin removal. Since cellulose has more hydroxyl groups (-OH) compared to hemicellulose and lignin [27], the O/C ratio increase in the WS-Alkali samples is greater than that in the WS-Acid samples. The high-resolution C 1s XPS spectra depicted in Figure 5b reveal the existence of four distinct types of carbon atoms, designated as C1–C4. These include C1: C-H, C-C; C2: C-O; C3: C=O, O-C-O; and C4: O-C=O. A careful analysis reveals that the C1 peak component, which is representative of lignin and extractives, exhibits a marked decrease in the WS-Alkali samples compared to both the WS-Acid and untreated WS samples. Conversely, the C2 peak component is significantly elevated in both the WS-Alkali and WS-Acid samples when compared to the WS samples. The decrease in the C1 peak component observed in the WS-Alkali samples is attributed to the alkali pretreatment, which effectively removed a significant portion of lignin and some hemicellulose from the wheat straw. Conversely, the dilute acid pretreatment removed only a fraction of the hemicellulose. Both pretreatment methods resulted in the exposure of increased amounts of cellulose within the wheat straw, which is rich in C-O bonds. This explains the elevation in the C2 peak components observed in the WS-Alkali and WS-Acid samples. These findings are in agreement with the O/C analysis. The XPS and high-resolution C 1s XPS spectra analysis provide valuable insights into the chemical composition of the hot-pressed wheat straw boards treated with different methods. Specifically, it is evident that alkali pretreatment effectively degrades a majority of lignin and some hemicellulose, while dilute acid pretreatment primarily degrades a portion of the hemicellulose.

### 3.3. Physical and Mechanical Properties Analysis

**Figure 6:**
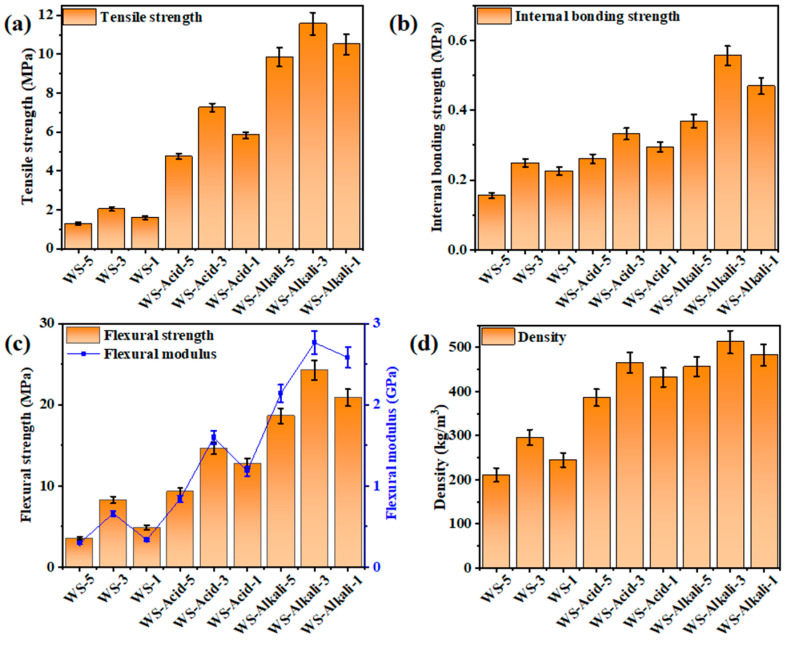
Physical and mechanical properties of hot-pressed wheat straw boards with different pretreatment methods and raw material sizes. (**a**) Tensile strength. (**b**) Internal bonding strength. (**c**) Bending strength and modulus of elasticity. (**d**) Water absorption rate.

The mechanical properties of hot-pressed wheat straw boards subjected to various pretreatment methods are presented in Figure 6a–d. A careful examination of Figure 6a indicates that as the particle size of the raw material decreases, the tensile strength of the boards exhibits a biphasic behavior, initially increasing and subsequently decreasing. Notably, the WS-Alkali-3 sample demonstrates superior tensile strength, achieving a peak value of 11.564 MPa. The analysis of Figure 6b reveals that the internal bonding strength of the boards exhibits a comparable trend, with the WS-Alkali-3 sample attaining a maximum value of 0.556 MPa. Furthermore, the bending strength and modulus of elasticity, as depicted in Figure 6c, mirror the patterns observed in tensile strength and internal bonding strength. Notably, the WS-Alkali-3 sample demonstrates superior performance in both bending strength and modulus, achieving peak values of 24.306 MPa and 2.766 GPa, respectively. The density of various board samples initially increases and subsequently decreases as the size of the raw materials varies. The WS-Alkali-3 sample exhibits the highest density among the tested samples, reaching up to 512 kg/m^3^. This phenomenon can be attributed to the enlarged specific surface area of the particles as their size diminishes, fostering tighter bonding between the wheat straw fibers. Nevertheless, excessively small particle sizes compromise the porosity of the board, leading to an uneven distribution of moisture and heat during the hot-pressing process. This, in turn, gives rise to an increase in micro defects within the board, ultimately resulting in a decline in its mechanical properties. Although both dilute acid and alkali pretreatments enhance the mechanical properties of the boards, the alkali pretreatment effect is more pronounced. This is due to several reasons. Firstly, alkali pretreatment effectively removes ash impurities from the surface of the wheat straw while simultaneously increasing the roughness of the wheat straw fiber surface, thereby facilitating compaction. Secondly, in comparison to dilute acid pretreatment, alkali pretreatment not only removes some hemicellulose but also a significant amount of lignin from the wheat straw. This process fully exposes the functional groups on the surface of cellulose, which favors the formation of chemical bonds during the hot-pressing process. Consequently, the self-adhesion strength of the board is strengthened. Furthermore, alkali pretreatment converts cellulose type I to type II, facilitating the formation of ether bonds through the condensation of hydroxyl groups in the amorphous regions. This process enhances the crystallinity of the board, leading to the reorganization of cellulose molecular chains during the hot-pressing process into a more stable crystalline structure. Analysis of the mechanical properties of the boards reveals that both alkali and dilute acid pretreatments effectively enhance the mechanical properties of hot-pressed wheat straw boards, with alkali pretreatment demonstrating superior effectiveness. Additionally, decreasing the size of the raw material particles further boosts the mechanical properties of the boards, albeit excessively small particle sizes may result in a decline in mechanical performance.

**Figure 7:**
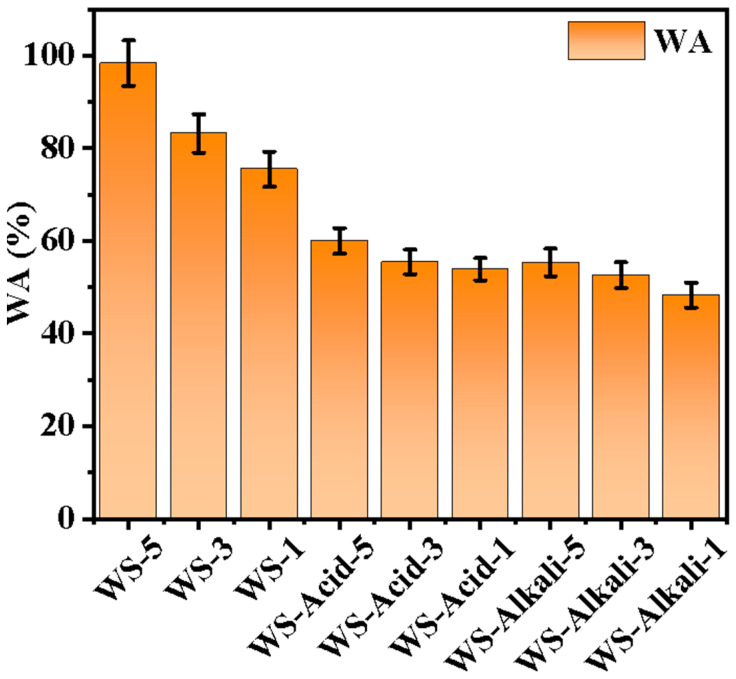
Water absorption rate of hot-pressed wheat straw boards with different pretreatment methods and raw material sizes.

Figure 7 presents the water absorption rates of hot-pressed wheat straw boards treated with various pretreatment methods. A comparison of the water absorption rates among the WS group samples reveals a consistent trend: as the size of the raw material particles decreases, the water absorption rate decreases regardless of the pretreatment method used. This observation suggests that reducing the size of the raw material particles contributes significantly to enhancing the water resistance of the boards. This is attributed to the increase in contact area between particles achieved by particle size reduction, which results in a more continuous and uniform structure during the hot-pressing process. Consequently, this reduction in particle size effectively decreases the porosity of the boards, further improving their water resistance properties. Lower porosity translates to fewer micro-pores, significantly slowing down the diffusion of moisture through the board’s microstructure, ultimately leading to a reduced water absorption rate. Notably, the WS-Alkali samples demonstrate the highest water resistance, attributable to the alkali pretreatment that effectively removes the majority of lignin and some hemicellulose from wheat straw. Since lignin exhibits low solubility in water, this pretreatment ensures improved water resistance in the resulting boards. The removal of lignin and hemicellulose through alkali pretreatment significantly contributes to the reduction of water absorption in the boards, rendering the WS-Alkali samples the most water-resistant. A thorough analysis of water absorption rates reveals that both alkali and dilute acid pretreatments can effectively enhance the water resistance of hot-pressed wheat straw boards. However, alkali pretreatment stands out as the more effective method for improving this critical property. Furthermore, a reduction in the size of raw material particles further bolsters the water resistance of the boards, underscoring the importance of particle size in optimizing board properties. While the performance of the boards we have prepared still lags behind commercial standards, the approach of pretreatment and controlling the size of raw material particles offers promising avenues for further enhancing their properties. This approach can be leveraged to selectively modify the surface morphology and functional groups of wheat straw, providing valuable insights and instructive suggestions for the development of agricultural waste boards.

The mechanical strength of the boards prepared in this study was compared with that in the literature and is shown in Table 1.

Upon comparing the mechanical strength, it can be seen that the boards prepared from reed straw subjected to NaOH pretreatment exhibited lower mechanical strength than the WS-Alkali-3 sample produced in this study. Meanwhile, boards derived from rice husk after hot-compressed water pretreatment demonstrated higher tensile strength than the WS-Alkali-3 sample; however, the hot-compressed water process is both complex and costly. Boards produced from jute following mechanical smashing and citric acid pretreatment, as well as those made from wheat straw and hemp straw via steam explosion pretreatment, exhibited superior internal bonding strength compared to the WS-Alkali-3 sample. Nonetheless, the cost of sourcing jute is considerably higher than that of wheat straw, and steam explosion pretreatment involves significant energy consumption.

The boards prepared in this study have natural degradability and environmental friendliness, can be applied to temporary building boards (such as sheds, motor houses, etc.) in the future, and can also be used for furniture boards (such as desktops and countertops).

### 3.4. Thermogravimetric Analysis

**Figure 8:**
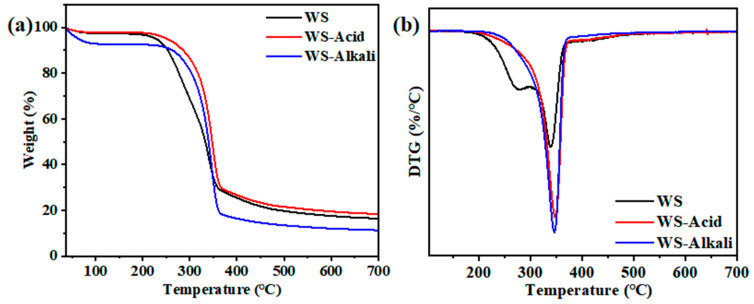
Thermogravimetric analysis of hot-pressed wheat straw boards with different pretreatment methods. (**a**) Thermogravimetric analysis (TG). (**b**) Differential thermogravimetric analysis (DTG).

Thermogravimetric analysis (TGA) serves as a reliable tool for assessing the thermal stability and decomposition behavior of materials. In Figure 8, the TGA results of hot-pressed wheat straw boards subjected to various pretreatment methods are presented. The WS-Alkali and WS-Acid samples demonstrate excellent thermal stability and degradation resistance during the combustion process. The occurrence of mass loss within a nitrogen-supplied, enclosed environment signifies a distinct, singular stage of thermal decomposition. The TG analysis in Figure 8a shows a sharp decline in thermal degradation of the board samples between 250 °C and 365 °C due to the thermal decomposition of lignin, cellulose, and hemicellulose [27]. The analysis reveals that the WS-Acid samples exhibit a higher initial degradation temperature compared to both the WS and WS-Alkali samples. Additionally, the weight loss rate of WS-Alkali samples is greater than that of WS and WS-Acid samples. This can be attributed to the fact that alkali pretreatment effectively removes the majority of lignin and some hemicellulose from wheat straw, whereas dilute acid pretreatment only removes a portion of the hemicellulose. The presence of lignin positively impacts the thermal stability of the boards, as it decomposes at higher temperatures [28]. On the contrary, alkali pretreatment results in the exposure of a greater number of functional groups, such as hydroxyl groups, which promote increased chemical reactions during the heating process. Consequently, the WS-Alkali samples exhibit a lower initial degradation temperature compared to the WS-Acid samples, while their weight loss rate is higher. As depicted in Figure 8b, the DTG analysis further confirms that the WS-Alkali samples display a higher maximum degradation rate than both WS and WS-Acid samples.

Therefore, WS-Acid samples exhibit superior thermal stability and resistance to thermal degradation during combustion. This is attributed to the fact that alkali pretreatment removes a significant amount of lignin and some hemicellulose, which are components that possess higher thermal stability. The removal of these components leads to an increased proportion of cellulose within the boards. Cellulose typically degrades over a narrower temperature range [29], leading to a higher maximum degradation rate on the DTG curve. Consequently, the TGA analysis reveals that WS-Acid samples possess greater thermal stability compared to the WS-Alkali samples.

### 3.5. Bonding Mechanism of Hot-Pressed Wheat Straw Boards

Compared to traditional glued boards, the hot-pressed wheat straw boards prepared in this study through dilute acid and alkali pretreatments eliminate the need for adhesives while maintaining excellent mechanical and physical properties. The bonding mechanism of these boards involves removing certain components, such as hemicellulose and proteins, from wheat straw through dilute acid pretreatment, which exposes a higher proportion of cellulose. The functional groups present on cellulose offer numerous bonding points during the hot-pressing process, enabling the intricate intertwining of wheat straw fibers. This interaction enhances the board’s self-adhesiveness.

Additionally, alkali pretreatment effectively removes the majority of lignin, along with some hemicellulose and proteins, further purifying the material. This process not only exposes more cellulose but also introduces additional chemically active sites, primarily hydroxyl groups. During hot pressing, these active sites react with the exposed functional groups on cellulose, creating new chemical bonds. This chemical interaction leads to a reorganization of the wheat straw fibers into a denser microstructure, thereby further strengthening the board’s self-adhesiveness.

The experiments were conducted under controlled laboratory conditions, which may not fully represent industrial-scale production. Besides, the corrosive impact of homogeneous acids and bases on equipment is an objective reality, and the utilization of solid acids and alkalis as pretreatment reagents warrants consideration in future research; however, the issue of cost remains a critical concern. Additionally, the long-term durability and environmental impact of the boards were not assessed. Future studies could focus on the environmental sustainability of these materials, including their biodegradability and long-term behavior under different environmental conditions. Exploring alternative, eco-friendly pretreatment methods or combining pretreatments to enhance board performance could be promising directions for continued research. Additionally, optimizing alkali pretreatment conditions to further improve performance and reduce processing costs, as well as investigating the boards’ long-term durability under varying conditions, would be valuable areas to explore.

## 4. Conclusions

This study delves into the fabrication of adhesive-free hot-pressed wheat straw boards, with a particular emphasis on exploring the impact of varying raw material sizes and acid/alkali pretreatments. The key findings of the study are as follows:

(i) The mechanical strength parameters of the optimal board sample obtained at 160 °C, 15 MPa, and 2 h hot-pressing parameters were 11.564 MPa (tensile strength), 0.556 MPa (internal bonding strength), 24.306 MPa, and 2.766 GPa (bending strength and modulus, respectively).
(ii) The investigation evaluated the impact of different raw material sizes (0.5 mm, 0.3 mm, and 0.1 mm) on the properties of hot-pressed wheat straw boards. The results showed that as particle size decreased, mechanical properties first improved and then declined, while water resistance consistently increased. The initial enhancement in mechanical properties was due to the larger specific surface area promoting tighter bonding among fibers. However, very small particles reduced porosity, causing uneven moisture and heat distribution during hot pressing and resulting in micro defects. The improved water resistance was attributed to a denser, more uniform structure with lower porosity, which slowed water diffusion and reduced absorption.
(iii) The impact of alkali and dilute acid pretreatments on hot-pressed wheat straw boards was examined, revealing that alkali pretreatment led to superior performance. This was due to the effective removal of lignin, some hemicellulose, and other components, which exposed cellulose functional groups, enhancing chemical bonding during hot pressing and boosting self-adhesive strength. Additionally, alkaline pretreatment transformed cellulose type I into type II, rearranging molecular chains and increasing crystallinity. In comparison, dilute acid pretreatment partially broke down hemicellulose and exposed the cellulose functional groups, also enhancing bonding. It disrupted crystalline regions, allowing amorphous cellulose to rearrange and increase crystallinity.

Typically, the pretreatment techniques for lignocellulosic biomass encompass chemical pretreatments such as steam explosion, heat and pressure reactor, microwaves, organosolv, and so on. The objective of this study was to explore the relationship between the chemical composition and properties, physical attributes like size, and the performance of the fabricated boards made of wheat straw raw materials from the perspective of their tissue structure. The influence and mechanism of acid and alkali pretreatment on the tissue structure level of lignocellulose are relatively well-established, facilitating the comparative discussion of our experimental results. Additionally, the cost of acids and alkalis is relatively low, and the maturity of the waste liquid treatment technology is high. Thus, the selection of acids and alkalis is also beneficial for the potential industrialization of this process.

## Figures and Tables

**Table 1:** Mechanical strength comparison.

Raw Material	Pretreatment Method	Tensile Strength (MPa)	Bending Strength (MPa)	Internal Bonding Strength (MPa)	Ref.
Wheat straw	Mechanical smashing and NaOH	11.564	24.306	0.556	This work
Reed straw	NaOH	/	18.2	0.45	[12]
Rice husk	Hot-compressed water	26	21	/	[13]
Jute	Mechanical smashing and citric acid	/	17.01	0.78	[14]
Wheat straw and hemp straw	Steam explosion	/	15.5	0.64	[15]

## Data Availability

Data are contained within the article.

## Supplementary Materials

The following supporting information can be downloaded at: https://www.mdpi.com/article/10.3390/ma17235845/s1, Figure S1: Photographs of hot-pressed wheat straw boards with different pretreatment methods and raw material sizes. (a) WS-5. (b) WS-3. (c) WS-1. (d) WS-Acid-5. (e) WS-Acid-3. (f) WS-Acid-1. (g) WS-Alkali-5. (h) WS-Alkali-3. (i) WS-Alkali-1.
