# Supplementary material for: Preparation of Hot-Pressed Wheat Straw Board by Self-Adhesive Process: Effects of Raw Material Sizes and Acid/Alkali Pretreatment
Source: Materials (Basel). 2024 Nov 28;17(23):5845. doi: 10.3390/ma17235845 (PMC11642262; doi:10.3390/ma17235845)
Supplement: Supplementary file 1 [file materials-17-05845-s001.zip › materials-3279596-supplementary.pdf]

Preparation of Wheat Straw Hot Pressing Board by Self Adhesive Process: Effects of Raw Material Sizes and Acid-Alkali Pretreatment

Jianing Wang [1], Ziyue Feng [1], Jiachen Zuo [1], Qinzhen Fan [1,*], Libo Zhang [1,2,*]

1 Guangdong Provincial Engineering & Technology Center for Corrosion and Safety in Petrochemical Industry, School of Chemical Engineering, Guangdong University of Petrochemical Technology, Maoming 525000, China; wangjianing62022@163.com (J.W.); zfengar@connect.ust.hk (Z.F.); zjcenormous@outlook.com (J.Z.)

2 State Key Laboratory of Heavy Oil Processing, College of Engineering, China University of Petroleum-Beijing at Karamay, Karamay 834000, China

* Correspondence: fanqinzhen@163.com (Q.F.); zhanglibo@cupk.edu.cn (L.Z.)

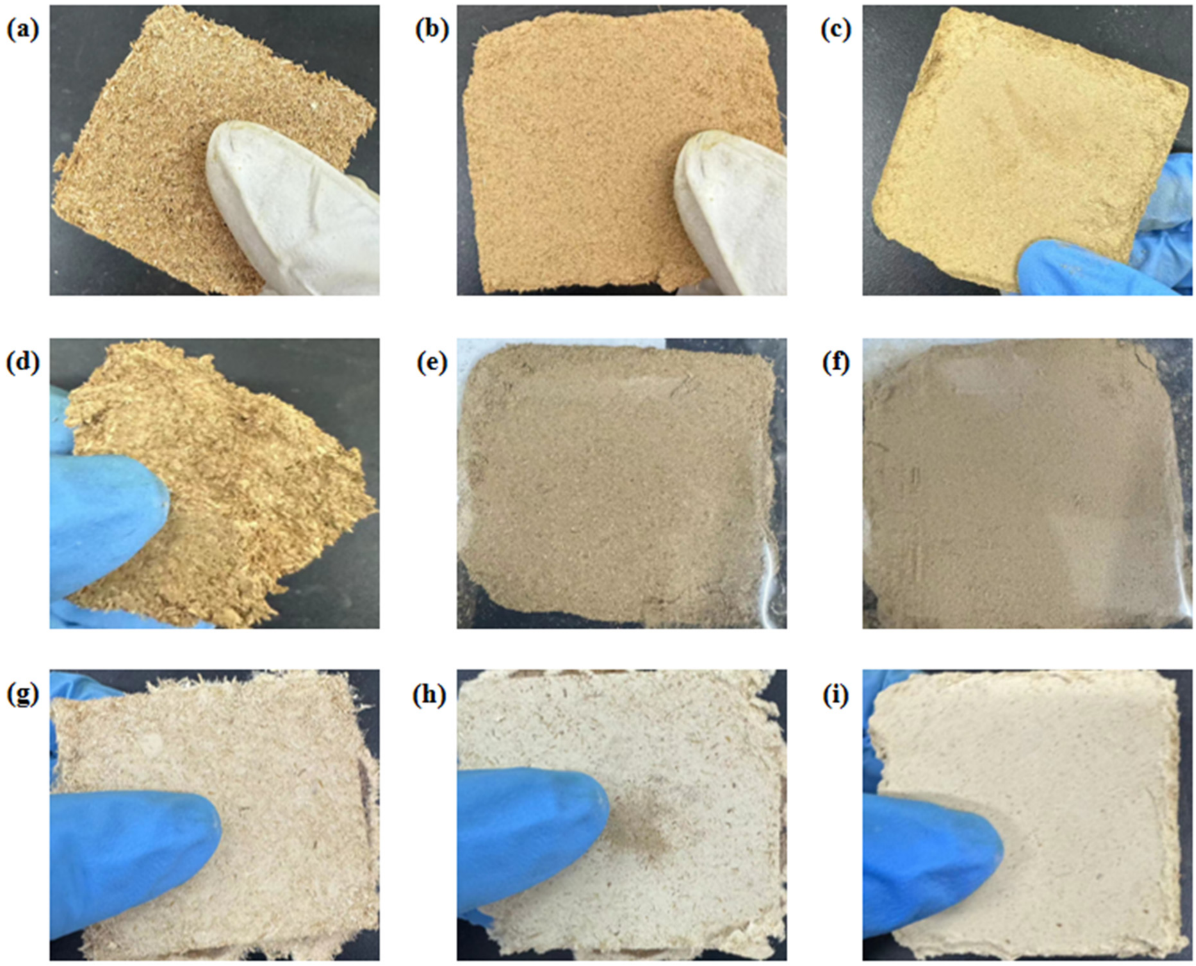

Figure S1. Physical photographs of wheat straw hot-pressed boards with different pretreatment methods and raw material sizes. (a) WS-5. (b) WS-3. (c) WS-1. (d) WS-Acid-5. (e) WS-Acid-3. (f) WS-Acid-1. (g) WS-Alkali-5. (h) WS-Alkali-3. (i) WS-Alkali-1.
